# Bacteria-induced expression of the pig-derived protegrin-1 transgene specifically in the respiratory tract of mice enhances resistance to airway bacterial infection

**DOI:** 10.1038/s41598-020-73084-2

**Published:** 2020-09-29

**Authors:** Fang Zeng, Chengcheng Zhao, Xiao Wu, Rui Dong, Guoling Li, Qingchun Zhu, Enqin Zheng, Dewu Liu, Jinzeng Yang, Stefan Moisyadi, Johann Urschitz, Zicong Li, Zhenfang Wu

**Affiliations:** 1 College of Marine Science, South China Agricultural University, Guangzhou, 510642 China; 2 National Engineering Research Center for Breeding Swine Industry, College of Animal Science, South China Agricultural University, Guangzhou, 510642 China; 3 Lingnan Guangdong Laboratory of Modern Agriculture, Guangzhou, 510642 China; 4 Department of Human Nutrition, Food and Animal Sciences, University of Hawaii at Manoa, Honolulu, HI USA; 5 Institute for Biogenesis Research, John A. Burns School of Medicine, University of Hawaii at Manoa, Honolulu, HI USA

**Keywords:** Genetic engineering, Genetic engineering

## Abstract

About 70% of all antibiotics produced in the world are used in the farm animal industry. The massive usage of antibiotics during farm animal production has caused rapid development of antibiotic resistance in bacteria, which poses a serious risk to human and livestock health when treating bacterial infections. Protegrin-1 (PG-1) is a potent antimicrobial peptide (AMP). It was initially identified in pig leukocytes with a broad-spectrum antibacterial and antiviral activity, and a low rate of inducing bacterial resistance. To develop a genetic approach for reducing the use of antibiotics in farm animal production, we produced transgenic mice carrying a bovine tracheal AMP gene promoter-controlled PG-1 transgene. The PG-1 transgene was specifically expressed in the respiratory tract of transgenic mice upon induction by bacterial infection. These PG-1 transgenic mice exhibited enhanced resistance to nasal bacterial infection as the transgenic mice showed a higher survival rate (79.17% VS. 34.78%), lower bacterial load and milder histological severity than their wild-type control littermates. The improved resistance to bacterial infection in the PG-1 transgenic mice could be resulted from the direct bacteria-killing activities of PG-1, and the immunomodulatory effects of PG-1 via stimulating interleukin 1 beta secretion. The present study provides a promising genetic strategy to prevent airway bacterial infections in farm animals by bacteria-inducible tissue-specific expression of PG-1 transgene. This approach may also be helpful for decreasing the possibility of inducing bacterial resistance during farm animal production.

## Introduction

About 70% of all antibiotics produced worldwide are used to improve the health and growth performance of farm animals^1^. In some countries, the amount of antibiotics used for farm animals accounts for approximately 80% of the nation’s total consumption^2^. This massive usage of antibiotics in food animals contributed significantly to the rapid development of antibiotic resistance, which is increasingly threatening the health of humans and animals^3–5^. Therefore, alternative methods that can reduce or replace the use of antibiotics in farm animal production are needed.

Antimicrobial peptides (AMPs) are a family of small polypeptides that are naturally expressed in many organisms as innate anti-infective agents^6,7^. AMPs, unlike traditional antibiotics, usually display low toxicity to non-target organisms and have little propensity for inducing bacterial drug resistance. AMPs also have a broad-spectrum defensive effect on various pathogens, including bacteria, fungi, viruses, and parasites^8–10^. Protegrin-1 (PG-1) is a potent AMP originally derived from pigs. It has a broad-spectrum antibacterial and antiviral activity^11–18^ and a low rate of inducing bacterial resistance^14^.

In an attempt to develop new strategies for reducing antibiotic consumption in farm animal production, transgenic mice expressing PG-1 were produced and these transgenic mice exhibited a significantly enhanced ability to fight against bacterial infections^19^. However, as the PG-1 transgene in these transgenic mice was driven by a constitutive promoter, which resulted in the constant and ubiquitous expression of PG-1, development of microbial resistance would be very likely. Therefore, promoters that are inducible only upon microbial infection and are active only in susceptible tissues should be used to control AMP transgene expression in transgenic animals. Respiratory diseases caused by airway microbial infection are devastating infectious diseases in the livestock industry that cause significant economic loss^20–22^. Therefore, using a respiratory tract-specific and bacterium-inducible promoter to drive AMP transgene expression could be an effective approach to improve resistance to infectious respiratory diseases in farm animals, while reducing the likelihood of inducing bacterial resistance.

In this study, we generated transgenic mice that carry a PG-1 transgene controlled by the bovine tracheal antimicrobial peptide (TAP) gene promoter. These transgenic mice were used as models to test whether the bacteria-inducible and respiratory tract-specific expression of PG-1 transgene in animals can increase the animals’ resistance to airway bacterial infection. The PG-1 transgenic mice showed specific expression of PG-1 in their respiratory tract upon induction of bacterial infection. These transgenic mice also exhibited enhanced resistance to nasal challenge of *Actinobacillus pleuropneumoniae* (A.pp) bacterium, which is the causative pathogen of the highly prevalent porcine pleuropneumonia disease^23^.

## Results

### Generation of PG-1 transgenic founder mice

**Figure 1:**
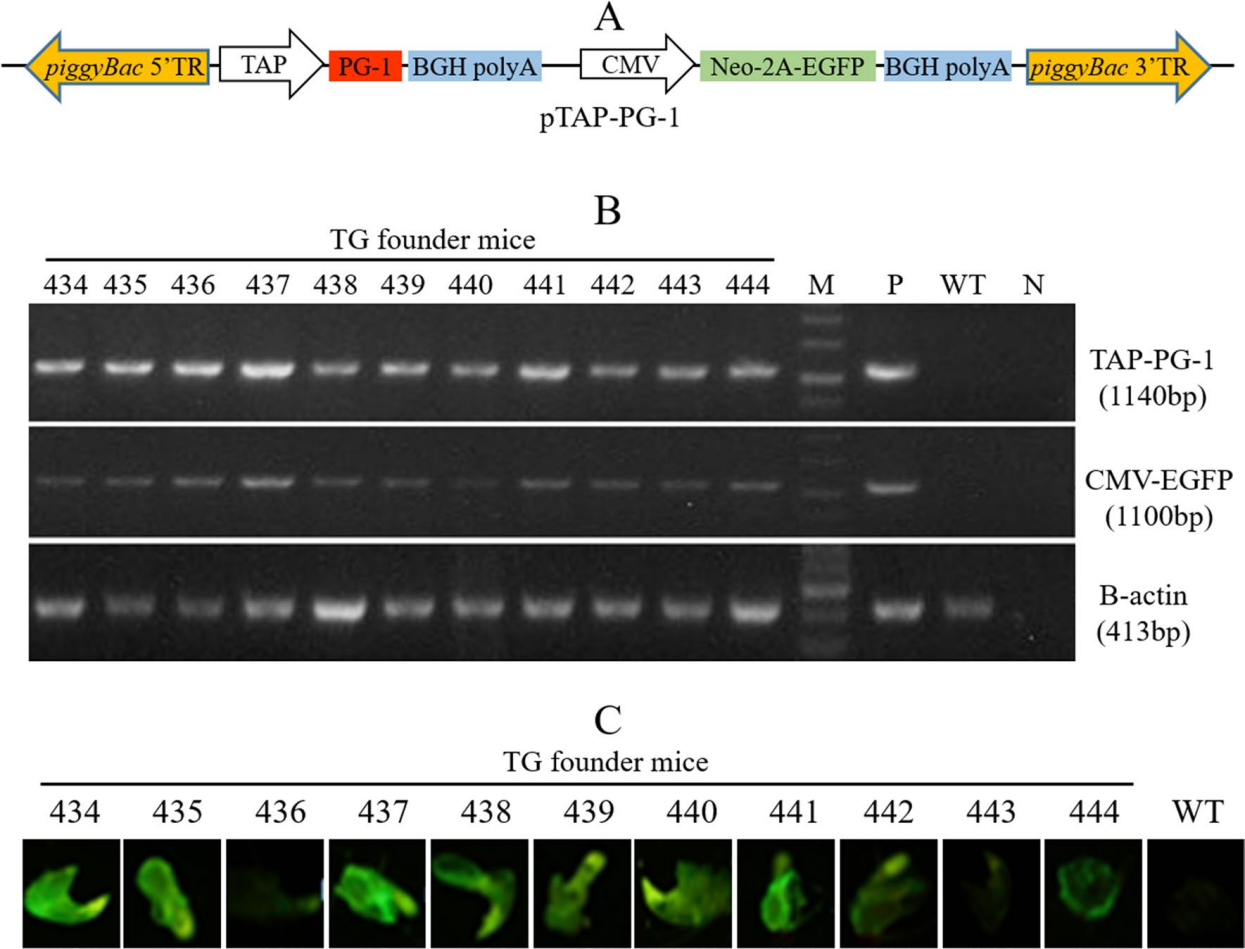
Generation of PG-1 transgenic mice. (**A**) Map of the constructed pTAP-PG-1 plasmid. The piggyBac 5′ and 3′ TRs, the piggyBac transposon 5′ and 3′ terminal repeat elements; TAP, the bovine trachea antimicrobial peptide gene promoter; PG-1, the pig-derived antimicrobial peptide protegrin-1 gene; BGH polyA, bovine growth hormone signal; CMV, cytomegalovirus promoter; Neo, the neomycin gene; 2A, the 2A peptide linker; EGFP, the enhanced green fluorescence protein gene. (**B**) PCR identification of PG-1 transgenic founder mice. M, marker; P, positive control using mixture of the pTAP-PG-1 plasmid and WT mouse DNA as template for PCR; WT, wild-type mice; N, negative control using water as template for PCR. β-Actin was amplified as internal control. (**C**) EGFP expression in the claw tissues of PG-1 transgenic founder mice. TG, transgenic mice. WT, wild-type mice.

A pTAP-PG-1 plasmid harboring a piggyBac transposon that carries a bacteria-inducible tracheal epithelial cell-specific bovine TAP promoter-driven PG-1 gene was constructed (Fig. 1A). This plasmid also contains a cytomegalovirus (CMV) promoter-controlled fusion selectable marker gene, which was composed of the neomycin (Neo) gene and an enhanced green fluorescence protein (EGFP) gene (Fig. 1A). The pTAP-PG-1 plasmid was co-injected with the piggyBac transposase expression plasmid pmPB^24^ into the pronuclei of mouse zygotes (C57BL/6 strain). Fifty-six pups were born following the transfer of 362 microinjected embryos into the oviducts of 12 surrogate mothers (ICR strain), and 11 born pups were identified as transgenic founder mice (Table 1). All 11 transgenic founder mice carried both the PG-1 and the EGFP gene in their genomes (Fig. 1B and Supplementary Fig. 1). EGFP expression, albeit at varied levels, was observed in the claw tissues of all 11 transgenic founder mice (Fig. 1C). No abnormal behavior or phenotype was detected in these 11 transgenic founder mice.

**Table 1:** Summary of the production of PG-1 transgenic mice by pronuclear microinjection.

No. of injected embryos	No. of transferred injected embryos	No. of surrogate mothers	No. of born mice	No. of transgenic mice
400	362	12	56	11

### Selection of transgenic mouse lines

**Figure 2:**
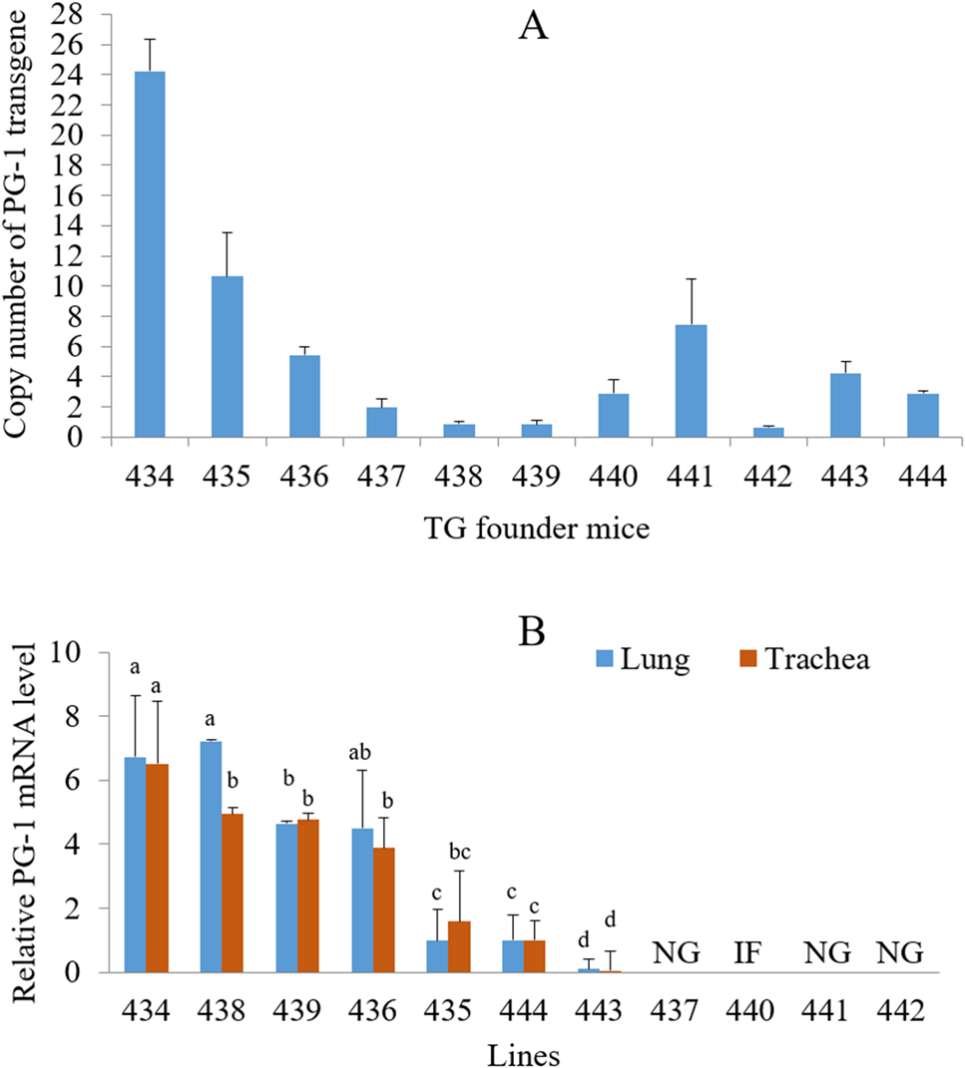
Selection of transgenic mouse lines. (**A**) Analysis of PG-1 transgene copy number in transgenic founder mice. Data shown are the means ± SEMs from triplicate experiments. (**B**) Comparison of PG-1 transgene expression level in the respiratory tract tissues among different transgenic mouse lines. Four to six transgenic mice were analyzed for each line, and data shown are the means ± SEMs. Values of the same tissue labeled with different lower case letters are significantly different at *P* *<* 0.05. *NG* no germline transmission of transgene, *IF* infertile.

Quantitative PCR (qPCR)-based transgene copy number analysis indicated that the copy number of the inserted PG-1 transgene varied from 1 to 24 in the 11 founders (Fig. 2A). Transgenic F_1_ offspring from the different founder lines expressed PG-1 mRNA at various levels in the trachea and lung tissues that contain tracheal epithelial cells (Fig. 2B). PG-1 transgene copy number was not positively correlated to its transcription level, as for example transgenic mice from a high transgene copy number line (line 434) and transgenic mice from two low transgene copy number lines (lines 438 and 439) expressed similar PG-1 mRNA levels (Fig. 2A,B). This finding could be due to the difference in the insertion sites of the randomly integrated transgene among different transgenic mouse lines. Transgenic F_1_ progenies from lines 434, 435, 436, 443, and 444 carrying multiple copies of PG-1 transgene exhibited larger variations in PG-1 mRNA expression levels than transgenic offspring from lines 438 and 439, which carried only one copy of PG-1 transgene (see the error bars in Fig. 2B). This phenomenon may have resulted from the segregation of the multiple copies of independently inserted transgenes after their transmission from the same line founder to its transgenic progeny. Therefore, line 438 transgenic mice were chosen for subsequent investigation as transgenic mice from this line carried only one copy of PG-1 transgene and yet expressed a relatively high level of PG-1 mRNA in their respiratory tract tissues (Fig. 2A,B).

### Characterization of transgene expression in transgenic progenies from line 438

**Figure 3:**
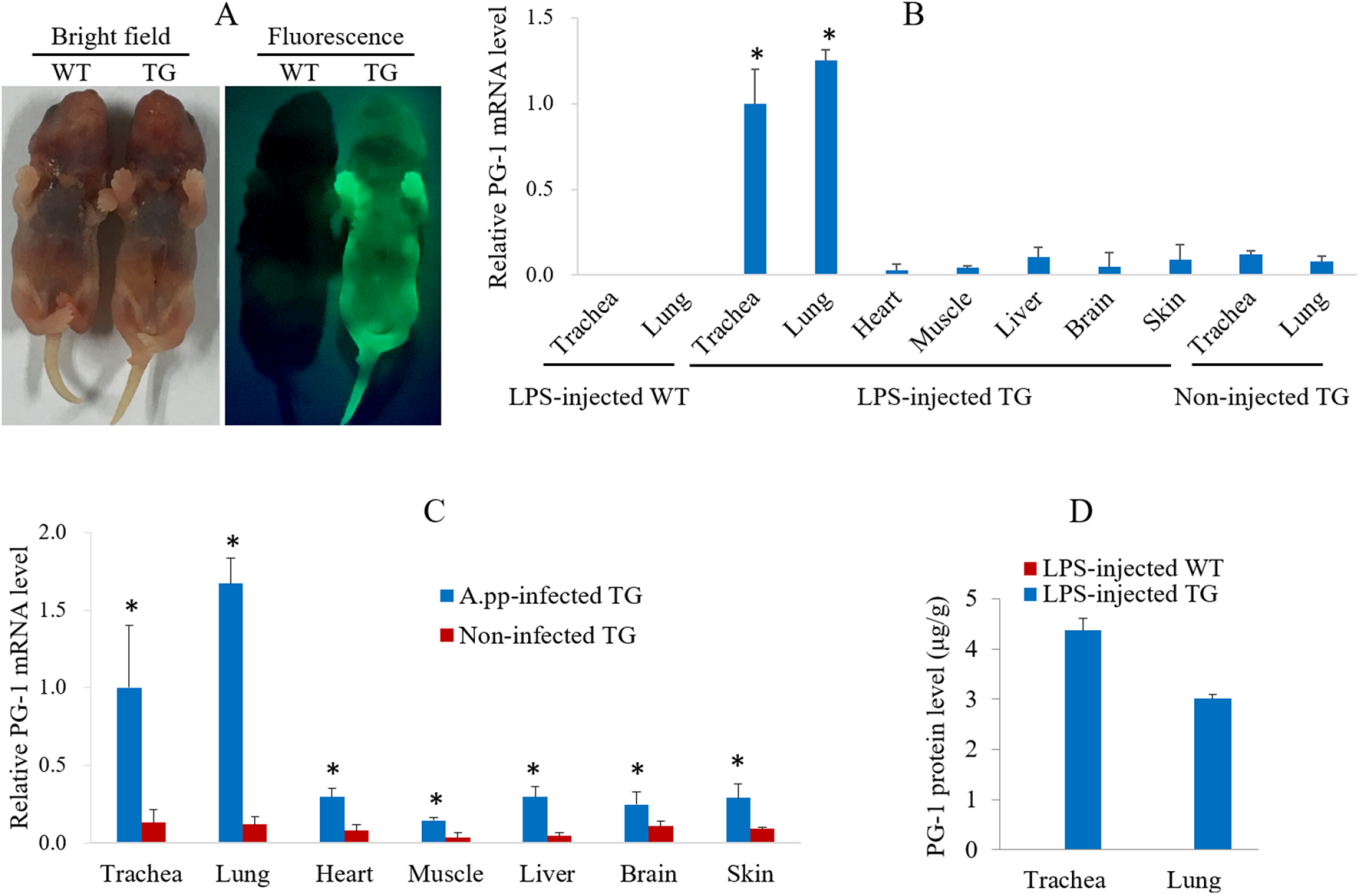
Characterization of transgene expression in transgenic mice from line 438. (**A**) Expression of EGFP marker gene in transgenic offspring from line 438. (**B**) Relative expression levels of PG-1 mRNA in the trachea, lung, heart, muscle, liver, brain, and skin tissues of line 438 transgenic mice with or without LPS treatment. (**C**) Relative expression levels of PG-1 mRNA in the trachea, lung, heart, muscle, liver, brain, and skin tissues of line 438 transgenic mice with or without A.pp infection. A.pp infection group’s tissue values labeled with a “*” means they are significantly different from the corresponding tissue value in no A.pp infection group. (**D**) PG-1 protein levels in the trachea and lung tissues of line 438 transgenic mice injected with LPS. Four transgenic mice were analyzed in B and C, and data shown are means ± SEMs.

Epifluorescence expression of EGFP was observed over the whole body of newborn transgenic progeny produced by mating transgenic founder 438 with wild-type (WT) mice (Fig. 3A). The PG-1 transcript levels in the trachea, lung, heart, muscle, liver, brain and skin tissues of line 438 transgenic mice injected with lipopolysaccharide (LPS) or A.pp were significantly higher than that in the same tissue of non-treated transgenic mice (Fig. 3B,C). This finding suggests that bacterial infection can induce expression of TAP promoter-driven PG-1 transgene in many different tissues of transgenic mice. However, bacterial infection-mediated induction resulted in a much higher PG-1 expression in the trachea and lung then in 5 other tested tissues (Fig. 3C). The PG-1 protein levels in the trachea and lung tissues of line 438 transgenic mice treated with LPS were 4.37 ± 0.23 and 3.01 ± 0.08 μg/g, respectively (Fig. 3D).

### PG-1 transgenic mice showed enhanced resistance to airway bacterial infection

**Figure 4:**
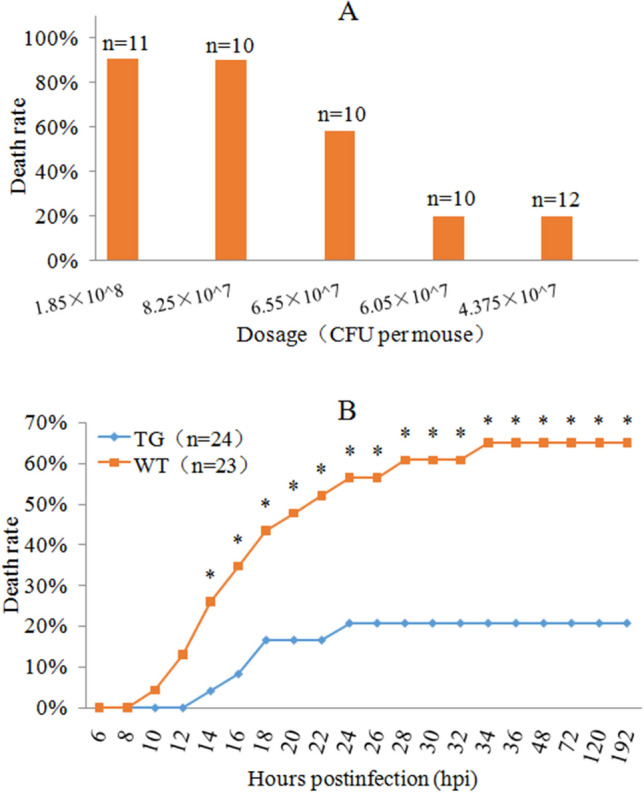
Analysis of resistance of PG-1 transgenic mice to nasal A.pp infection. (**A**) Valuation of susceptibility of WT mice to A.pp infection by nasal inoculation of the different A.pp dosages. Infected mice were monitored for 8 days (192 h) to calculate death rate. (**B**) Comparison of death rate between line 438 transgenic mice and their WT littermates after nasal inoculation of A.pp at 6.555 × 10^7^ CFU per mouse. *Mean difference between two groups reaches level of significance at *P* < 0.05.

Susceptibility testing demonstrated that nasal inoculation of A.pp at a dosage of 6.55 × 10^7^ colony forming unit (CFU) per mouse resulted in a 58.3% death rate in WT mice (Fig. 4A). Therefore, this dosage is close to the median lethal dose and was used as the nasal challenge dosage to compare the resistance to A.pp infection between transgenic and WT mice. After nasal infection with A.pp at a dosage of 6.55 × 10^7^ CFU/mouse, typical clinical signs such as labored breathing, ruffled hair coat, lethargy and hunched posture, were seen after about 5 h post infection (hpi), and death was observed at 10 hpi in infected WT mice and 14 hpi in infected transgenic mice. The death rate of A.pp-challenged PG-1 transgenic mice was substantially lower than that of their infected WT littermates from 14 to 192 hpi, and the overall mortality rates of transgenic and WT groups were 20.83% and 65.22%, respectively, during the monitoring period of 192 hpi (Fig. 4B).

**Figure 5:**
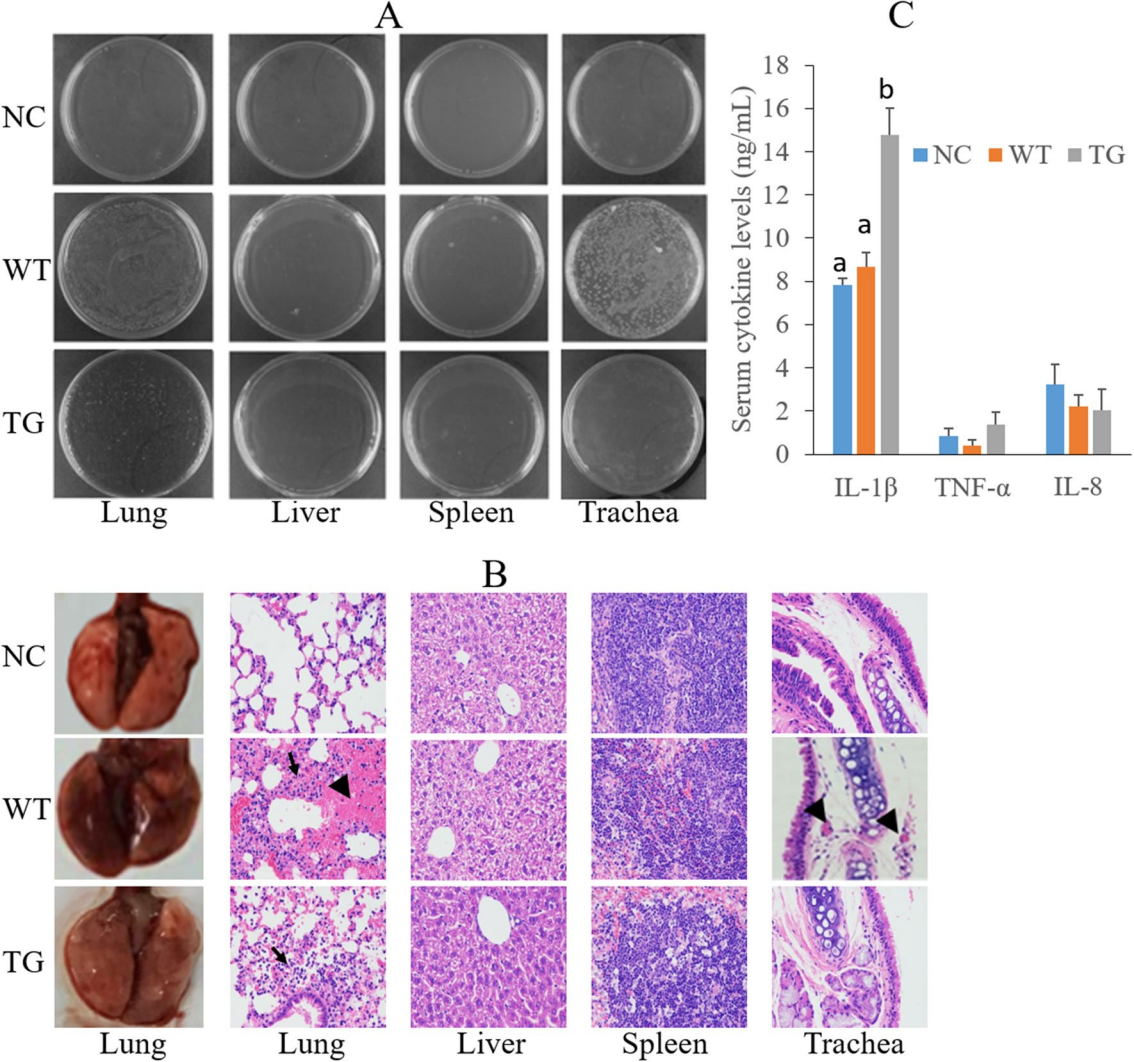
Comparison of A.pp bacterial load, histological features, and serum cytokine concentrations among surviving transgenic (n = 4) and WT (n = 4) mice at 6 hpi and non-infected NC mice (n = 3). (**A**) Representative culture plates of A.pp colonies recovered from homogenized tissues of surviving transgenic and WT mice at 6 hpi and non-challenged NC mice. (**B**) Histological analysis of surviving transgenic and WT mice at 6 hpi and non-challenged NC mice. Representative sections are shown. Lung and trachea sections show that WT mice have severe focal congestion (indicated by arrow head), which was not found in the lungs of NC mice and challenged transgenic mice. Lung sections indicate that the alveolar spaces are clear in NC mice but have mild and severe neutrophilic infiltrates (indicated by arrows) in transgenic and WT mice, respectively. Liver and spleen sections show that all groups of mice have similar features. (**C**) Serum cytokine concentrations in surviving transgenic and WT mice at 6 hpi and non-challenged NC mice. Values labeled with different superscripts are significantly different at *P* < 0.01.

At 6 hpi, no A.pp bacteria were recovered from the liver and spleen of surviving WT and transgenic mice challenged by nasal inoculation, but the number of A.pp bacteria recovered from the lung and trachea of infected WT mice was higher than that of their transgenic littermates (Fig. 5A). Severe hemorrhage in the lung was observed in infected WT mice but not in infected transgenic mice or non-infected negative control (NC) mice at 6 hpi (Fig. 5B). Infected WT and transgenic mice showed normal features in the liver and spleen sections. Infected transgenic mice exhibited mild neutrophilic infiltrate while infected WT mice exhibited severe neutrophilic infiltrate and congestion in the lung sections compared with NC mice at 6 hpi (Fig. 5B). Congestion was observed in the tracheal sections of WT mice but not in transgenic mice at 6 hpi (Fig. 5B). The serum concentration of IL-1β, which is a PG-1-regulated cytokine^25^, was higher in infected transgenic mice than in NC mice and challenged WT mice at 6 hpi (Fig. 5C). However, the serum level of two other cytokines, including TNF-α and IL-8, was similar among NC, infected WT and transgenic mice at 6 hpi (Fig. 5C).

An analysis of blood collected at 6 hpi indicated that mean corpuscular volume (MCV), mean corpuscular hemoglobin concentration (MCHC) and red cell distribution width-coefficient of variation (RDW-CV) were similar between WT and transgenic mice, but these three blood indexes were lower in infected WT and transgenic mice than those in NC mice (Table 2). All other blood parameters measured at 6 hpi were similar among the three groups of mice except the neutrophil number (Neu#) in transgenic mice was higher than those in NC and WT mice (Table 2).

**Table 2:** Comparison of routine blood parameters among non-infected NC, A.pp-infected WT, and A.pp-infected transgenic live mice at 6 hpi.

	NC (n = 6)	WT (n = 4)	TG (n = 4)
WBC# (10^9^/L)	3.77 ± 0.83	3.52 ± 0.64	3.51 ± 0.81
Bas# (10^9^/L)	0.022 ± 0.01	0.01 ± 0.000	0.018 ± 0.015
Neu# (10^9^/L)	0.89 ± 0.43^A^	0.57 ± 0.19^A^	1.13 ± 0.26 ^B^
Eos# (10^9^/L)	0.008 ± 0.009	0.005 ± 0.006	0.015 ± 0.006
Lymph# (10^9^/L)	2.84 ± 0.81	2.77 ± 0.41	1.95 ± 0.67
Bas (%)	0.62 ± 0.26	0.58 ± 0.25	0.65 ± 0.27
Neu (%)	23.87 ± 12.46	28.17 ± 9.70	36.85 ± 8.36
Eos (%)	0.23 ± 0.19	0.30 ± 0.25	0.50 ± 0.16
Lymph (%)	75.23 ± 12.52	70.95 ± 9.68	61.95 ± 8.58
RBC (10^12^/L)	8.45 ± 1.54	9.17 ± 1.30	9.56 ± 0.24
HGB (g/L)	128.67 ± 15.01	142.75 ± 17.89	147.75 ± 6.18
MCV (fL)	49.57 ± 0.27 ^A^	48.45 ± 0.95^B^	47.98 ± 0.46^B^
MCH (pg)	15.20 ± 0.34	15.65 ± 0.42	15.43 ± 0.34
MCHC (g/L)	306.50 ± 7.04^A^	322.50 ± 2.38^B^	321.75 ± 4.99^B^
RDW-CV (%)	13.15 ± 0.50^A^	12.25 ± 0.60^B^	11.83 ± 0.10^B^
RDW-SD (fL)	25.85 ± 0.87	23.58 ± 0.99	22.75 ± 0.13
HCT (%)	41.92 ± 7.64	44.35 ± 5.61	45.88 ± 1.50
PLT (10^9^/L)	703.17 ± 212.09	620.00 ± 214.11	747.75 ± 194.51
MPV (fL)	6.38 ± 0.89	5.68 ± 0.36	5.83 ± 0.43
PDW (%)	15.10 ± 0.14	14.97 ± 0.13	15.05 ± 0.58
PCT (%)	0.43 ± 0.09	0.35 ± 0.11	0.43 ± 0.10

WBC#, white blood cell number; Bas#, basophil number; Neu#, neutrophil number; Eos#, eosinophil number; Lymph#, lymphocyte number; Bas%, percentage of basophils; Neu%, percentage of neutrophils; Eos%, percentage of eosinophils; Lymph%, percentage of lymphocytes; RBC, red blood cell number; HGB, hemoglobin concentration; MCV, mean corpuscular volume; MCH, mean corpuscular hemoglobin; MCHC, mean corpuscular hemoglobin concentration; RDW-CV, red cell distribution width-coefficient of variation; RDW-SD, red cell distribution width-standard deviation; HCT, hematocrit; PLT, platelet number; MPV, mean platelet volume; PDW, platelet distribution width; PCT, plateletcrit. Values in the same row labelled with different superscript are significantly different at *P* *<* 0.05.

Genomic DNA of recovered bacteria was subjected to PCR amplification for an A.pp bacteria-specific gene (APXIVA) followed by sequencing of the PCR products, which confirmed that the bacteria recovered from the lung, liver, spleen, and trachea tissues of challenged transgenic and WT mice were derived from the inoculated A.pp (data not shown).

## Discussion

In this study, we produced transgenic mice carrying a PG-1 transgene under the control of a bacteria-inducible and respiratory tract tissue-specific promoter. These PG-1 transgenic mice were healthy and predominately fertile. In comparison with their WT littermates, they exhibited enhanced resistance to A.pp bacterial infection as evidenced by their higher survival rate, lower tissue bacterial load, and milder histological severity after A.pp inoculation. These results were similar to those reported in the transgenic mice that exhibited ubiquitous expression of PG-1 under the control of a CMV promoter^19^. Yet, with the transgenic mice generated in the present study the likelihood of inducing bacterial resistance to antibiotics is further reduced, as PG-1 expression is restricted to the respiratory tract only upon induction by bacterial infection. Expressions of many AMPs in their native host animals are induced via microbial infection and are tissue-specific^26–28^. This phenomenon could be the evolution outcome to minimize the chances of inducing microbial resistance. Therefore, if an AMP transgene is used to defense bacterial infections in animals, its expression should be controlled by a bacteria-inducible and tissue-specific promoter. Although wild-type pigs carry a PG-1 gene in their genome, it is naturally expressed in leukocytes^12^. In the future, transgenic pigs overexpressing PG-1 in their respiratory tract can be produced to investigate whether their resistance to airway bacterial infections is improved.

Some AMPs not only directly target and destroy bacteria, but also indirectly inhibit bacterial infection by regulating the host immune responses via several different approaches, such as enhancing proinflammatory cytokine secretion and recruiting immune cells to the infection sites^29–33^. PG-1 has direct bacteria-killing activities^34–39^ and also participates in immune modulation by stimulating the rapid and efficient release of mature IL-1β from monocytes^25^. Our findings are in agreement with these reports, as serum IL-1β levels were higher in our transgenic mice than those in WT and NC mice at 6 hpi. However, the PG-1 transgene might only have a mild immunomodulatory effect in transgenic mice because the serum level of two other cytokines, TNF-α and IL-8, was not elevated in infected transgenic mice at 6 hpi, compared with NC and infected WT mice. In addition, our observation that PG-1 transgenic mice have higher numbers of neutrophils than WT or NC mice at 6 hpi is consistent with a previous study, which reported that PG-1 to exert immune modulatory effects via promoting neutrophil migration to the infection sites to reduce bacterial colonization^19^. The increased neutrophil number might be related to the elevated serum IL-1β level in PG-1 transgenic mice since many studies showed that neutrophils can secret IL-1β through the NLPR3 inflammosome^40–42^, which plays vital roles in releasing IL-1β from cells^43,44^.

Expression of the TAP promoter-controlled PG-1 transgene increased the transgenic mice’s resistance to nasal A.pp infection. However, whether its expression can also enhance the ability of transgenic animals against other important airway bacterial infections remains to be determined. Therefore, future studies are needed to assess if the PG-1 transgenic mice produced in this study are also resistant to other respiratory tract pathogens, such as A. suis^45,46^ and Streptococcus suis^47^, which also cause serious contagious diseases in farm animals. The PG-1 transgenic mice also can be used to test whether their resistance to airway viral infections is improved, because many AMPs, including PG-1, have been shown to effectively inhibit both bacterial and viral infection^8–10^. In addition, the PG-1 transgenic mice created in the present study provide a valuable animal model for investigating the in vivo function and mechanism of action of PG-1.

In summary, expression of PG-1 under the control of the TAP promoter enhanced the resistance of transgenic mice to airway bacterial infection. The bacteria-inducible and respiratory tract-specific expression of PG-1 transgene is a promising genetic strategy to control airway bacterial infections in farm animals. This strategy may also be helpful for decreasing the chance of inducing bacterial resistance during farm animal production.

## Materials and methods

### Ethics statement

This study was conducted in strict accordance with ‘‘Guidelines with Respect to Caring for Laboratory Animals’’ issued by the Ministry of Science and Technology of China. The animal experimental protocol was approved by the Institutional Animal Care and Use Committee of South China Agricultural University. All efforts were made to minimize animal suffering.

### Bacterial strain

A.pp serotype 1 bacteria were purchased from the China Veterinary Culture Collection Center (catalog no: CVCC259, Beijing, China). A.pp was cultured on tryptic soy agar (TSA) or in tryptic soy broth (TSB) (Difco Laboratories, USA) supplemented with 10 µg/ml of nicotinamide adenine dinucleotide (NAD) and 10% (v/v) filtered bovine serum at 37 °C as described previously^48^.

### Plasmid construction

A 890-bp DNA fragment containing the bacteria-inducible tracheal epithelial cell-specific bovine TAP promoter^49–51^ and the 450-bp pig PG-1-coding sequences (GenBank Accession no: X79868.1) was synthesized by the GENEWIZ Company (Suzhou, China). This fragment was used to replace the PSP-hNGF fragment between the Age I and Asc I sites of the pmPSP-hNGF plasmid^52^, to generate the pTAP-PG-1 plasmid. The DNA sequences of pTAP-PG-1 plasmid were confirmed by sequencing.

### Generation of transgenic mice

The pTAP-PG-1 plasmid and the piggyBac transposase expression plasmid pmPB were co-injected into the pronucleus of one-cell-stage mouse embryos (C57BL/6 strain), which were then transferred into the oviducts of Institute of Cancer Research (ICR) strain surrogate mothers. The recipient females were mated with vasectomized stud males of ICR strain the day before embryo transfer. Pregnant recipient mothers were allowed to deliver and raise their pups.

### PCR identification of transgenic mice

PCR identification of transgenic mice was performed as previously reported^53^. Genomic DNA was isolated from the tail tissues of founder mice by using the tissue DNA extraction kit (Omega, Doraville, USA). The TAP-PG-1 transgene, CMV-EGFP marker gene, and the β-actin internal control gene were amplified by PCR. The PCR amplification products were sequenced to confirm their identities.

### Observation of EGFP expression

Observation of EGFP expression was performed as previously reported^53^. EGFP expression in the claw tissues of founder transgenic mice was observed under the fluorescence microscopy. EGFP expression in newborn line 438 transgenic mice was visualized by the Living Organism’s fluorescent protein observation system (Model: FBL, BLS company, Budapest, Hungary).

### qPCR analysis of transgene copy number

PG-1 transgene copy number was analyzed as previously reported^54^. A standard set of mixtures of pTAP-PG-1 plasmid DNA with WT mouse genomic DNA representing 1, 10, 100, 1000, 10,000, and 100,000 copies of PG-1 transgene per mouse genome was prepared. The mixture from each standard sample at 2 µl was used as template for qPCR measurement of threshold cycle (Ct) values. A standard curve was established based on the measured Ct values of all the standard samples and their corresponding transgene copy number. The Ct values of all transgenic founder mice’s tail genomic DNA were also measured by qPCR following the same protocol. The Ct value of each transgenic founder mouse genomic DNA was converted into corresponding PG-1 transgene copy number based on the established standard curve.

### qPCR analysis of transgene expression

Transgenic mice were injected intraperitoneally with 15 μg of LPS from Salmonella typhimurium (Catalog no. L-7261, Sigma) to induce PG-1 transgene expression. Total RNA was extracted from the collected tissues of injected transgenic mice by E.Z.N.A. total RNA kit I (OMEGA, Doraville, GA, USA). cDNA was synthesized by PrimeScript RT reagent kit with gDNA Eraser (Takara, Dalian, China). The Ct values of PG-1 and internal control glyceraldehyde phosphate dehydrogenase were analyzed by qPCR via the Eco real-time PCR system (Illumina, San Diego, CA, USA) and SYBR Premix Ex Taq (Takara, Dalian, China). Relative transgene mRNA level was calculated by the 2^−ΔΔCt^ method. The qPCR products were sequenced to confirm their identities.

### ELISA analysis of protein concentration

The PG-1 protein concentration in the tissues was analyzed by enzyme-linked immunosorbent assay (ELISA) kit for pig PG-1 (catalog no: E1705p, EIAab, Wuhan, China) by following the manufacturer’s instructions. Serum cytokine concentrations were measured by mouse IL-1β ELISA kit (Boster Biotechnology Co., Ltd., Wuhan, China), TNF-α ELISA Kit (Beyotime company, Shanghai, China) and IL-8 ELISA Kit (Solarbio company, Beijing, China) according to the manufacturer’s instructions.

### A.pp challenge and post challenge monitoring

Transgenic mice and their WT littermates from line 438 were identified by PCR and confirmed by the observation of EGFP expression on their claw tissue. All the mice were housed in a pathogen-free and temperature-controlled room with 12 h light/12 h dark cycle. They were raised independently in their own cages with free access to food and water. Serological testing was performed before the A.pp infection to confirm that all the animals were free of usual viral and bacterial pathogens. The concentration of cultured A.pp (CFU/ml) was determined by the standard curve method via measuring the optical density value at 600 nm. Mice were challenged by nasal drop of 20 µl of A.pp, and monitored every 2 h to determine their status of survival or death.

### Blood parameter analysis

Blood parameter analysis was performed as previously described^53^. Blood (200–400 µl) was collected into EDTA-containing tubes from the retro-orbital plexus of surviving mice at 6 hpi of A.pp. Collected blood samples were stored at 4 °C and analyzed through an automatic hematology analyzer within 1 h.

### Bacterial load measurement

Bacterial load measurement was performed as previously described^53^. Surviving mice were euthanized at 6 hpi. Tissues (approximately 10 to 40 mg) were collected immediately after euthanization and placed in pre-weighed sterile 2-ml tubes. The weight of each tissue was measured, and sterile phosphate-buffered saline (PBS) was added to each tissue sample at a ratio of 10 µl of sterile PBS to 1 mg of tissue. All samples were immediately homogenized with a homogenizer. The resulting tissue homogenate was spread on three 100 µl TSA plates. The plates were incubated overnight at 37 °C in an atmosphere of 5% CO_2_. Bacteria showing the characteristic A.pp phenotype were counted.

### Histological analysis

Histological analysis was performed as previously described^53^. Tissues collected from the euthanized mice at 6 hpi were gently instilled with 10% buffered formalin, immersed in the same solution for fixation, embedded in paraffin, sectioned, and stained with hematoxylin–eosin. The stained slides were examined under a microscope.

### PCR identification of A.pp

PCR identification of A.pp was performed as previously described^53^. Bacterial colonies recovered from tissues of challenged transgenic and WT mice at 6 hpi were isolated from the TSA plates and grown in TSB supplemented with 10 µg/ml of NAD and 10% (v/v) filtered bovine serum at 37 °C for 8 h. One microliter of bacteria-containing medium was used as template for PCR to amplify a gene in the genome of A.pp. The PCR product was sequenced, and the sequencing result was blasted against A.pp genomic DNA.

### Statistical analysis

Chi-square test was used to determine differences in survival rate between the two groups, whereas student t-test was used to compare differences in tissue PG-1 mRNA level and serum IL-1β concentration between the two groups. Significant difference was determined at *P* < 0.05.

## Supplementary information

Supplementary Figure S1.

## Data Availability

The datasets used and analyzed during the current study are available from the corresponding author on reasonable request.
