# Supplementary material for: Bacteria-induced expression of the pig-derived protegrin-1 transgene specifically in the respiratory tract of mice enhances resistance to airway bacterial infection
Source: Sci Rep. 2020 Sep 29;10:16020. doi: 10.1038/s41598-020-73084-2 (PMC7524760; doi:10.1038/s41598-020-73084-2)

**Bacteria-induced expression of the pig-derived protegrin-1 transgene specifically in the respiratory tract of mice enhances resistance to airway bacterial infection**

Fang Zeng^1, 2,^ ^3, ¶^, Chengcheng Zhao^2, 3, ¶^, Xiao Wu^2, 3^, Rui Dong^2, 3^, Guoling Li^2, 3^, Qingchun Zhu^2, 3^, Enqin Zheng^2, 3^, Dewu Liu^2, 3^, Jinzeng Yang^4^, Stefan Moisyadi^5^, Johann Urschitz^5^, Zicong Li^2, 3, *^, Zhenfang Wu^2, 3, *^

^1^College of Marine Science, South China Agricultural University, Guangzhou, China, 510642

^2^National Engineering Research Center for Breeding Swine Industry, College of Animal Science, South China Agricultural University, Guangzhou, China, 510642

^3^Lingnan Guangdong Laboratory of Modern Agriculture, Guangzhou, China, 510642

^4^Department of Human Nutrition, Food and Animal Sciences, University of Hawaii at Manoa, Honolulu, Hawaii, USA

^5^Institute for Biogenesis Research, John A. Burns School of Medicine, University of Hawaii at Manoa, Honolulu, Hawaii, USA

^¶^Fang Zeng and Chengcheng Zhao contributed equally to this work.

^*^Address correspondence to Zicong Li (email: lizicong@scau.edu.cn) and Zhenfang Wu (email: wzfemail@163.com)

Supplementary figure 1:


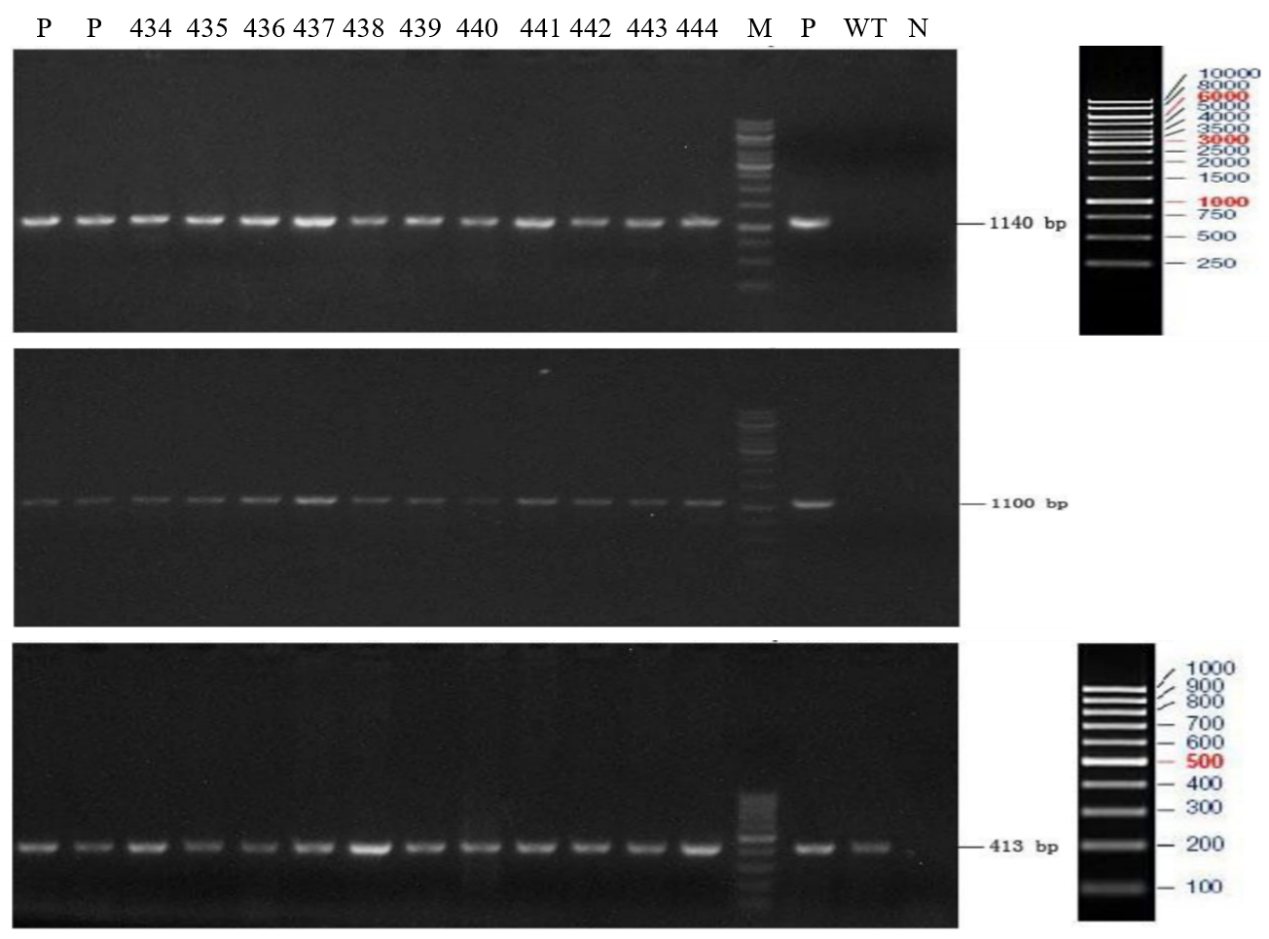

Supplement: Supplementary file 1 — Supplementary Figure S1. [file 41598_2020_73084_MOESM1_ESM.docx]
